# Experiences of Dental Care and Dental Anxiety in Adults with Autism Spectrum Disorder

**DOI:** 10.1155/2014/238764

**Published:** 2014-10-28

**Authors:** My Blomqvist, Göran Dahllöf, Susanne Bejerot

**Affiliations:** ^1^Division of Paediatric Dentistry, Department of Dental Medicine, Karolinska Institutet, 141 04 Huddinge, Sweden; ^2^Department of Clinical Neuroscience, Karolinska Institutet and Northern Stockholm Psychiatry, St. Göran Hospital, 112 81 Stockholm, Sweden

## Abstract

Dental anxiety is associated with previous distressing dental experiences, such as lack of understanding of the dentist intentions, perceptions of uncontrollability and experiences of pain during dental treatment. People with autism spectrum disorder (ASD) are impaired in building flexible predictions and expectations, which is very much needed during a dental visit. The aims of the study were to investigate if people with ASD have more negative dental experiences and a higher level of dental anxiety compared to a matched control group. Forty-seven adults with ASD and of normal intellectual performance, and 69 age- and sex-matched typically developing controls completed questionnaires on previous dental experiences and dental anxiety, the Dental Anxiety Scale, and the Dental Beliefs Survey. The ASD group experienced pain during dental treatments more often than the controls and 22% had repeatedly experienced being forced to dental treatment they were not prepared for, compared to 3% of the controls. A higher level of dental anxiety was reported by the ASD group. Dental treatment and methods for supporting the communication with patients with ASD need to be developed, in order to reduce the negative dental experiences and dental anxiety in people with ASD.

## 1. Introduction

Dental anxiety, affecting approximately 20% of the adult population [1], is a feeling that something dreadful is going to happen in relation to dental treatment and is connected to a sense of losing control [2]. It is associated with dental caries and missed appointments [3, 4] and may extend to a total avoidance of dental care, reported by approximately 5% of the general population [1]. The development of dental anxiety is associated with previous distressing dental experiences, such as lack of understanding of the dentist intentions, or feeling of extreme helplessness or severe embarrassment during the treatment [5]. There is also a strong relationship between the number of previous experiences of pain during dental treatments and dental anxiety [6]. In children, both dental fear and pain connected to dental injections is more frequently reported in girls [7, 8]. Furthermore, medical treatments or conditions, other than dental, that might have caused pain, are associated with dental anxiety, at least in children [9]. However, Armfield [10] argued that it is the person's perception of the situation rather than the pain itself that is crucial for the development of dental anxiety. Perceptions of uncontrollability and feelings of danger have both showed strong associations with a high prevalence of dental anxiety [11].

Autism spectrum disorder (ASD) is considered to be a neurodevelopmental condition with early childhood onset [12]. It is characterised by persistent impairments in social interaction and communication and restricted, repetitive patterns of behaviour, interests, or activities and unusual sensory interests or sensitivities. In the recently revised American classification system for psychiatric disorders, the DSM-5 [12], ASD now includes previously separate diagnostic categories such as autistic disorder, Asperger disorder, and pervasive developmental disorder, not otherwise specified, specified (PDD-NOS) in the DSM-IV-TR [13] (American Psychiatric Association, 2000).

People with ASD commonly report abnormal responses to sensory stimuli [14]. Overreactivity to sound and under-reactivity to pain was stated by more than 40% of children with ASD. Sensory abnormalities affected to a greater extent those with more severe autistic traits compared to those with less autistic traits [14]. Sensory sensitivities in children with ASD are also related to behaviour difficulties in the dental office [15]. There are yet no studies on behaviour and experiences of dental care in adults with ASD.

People with ASD are impaired in building flexible predictions and expectations, which is very much needed in the highly unpredictable social world [16]. An inability to expect new sensory inputs and events leads to difficulties in perceptions and executive functions such as flexibility and planning [16] and this stress presumably causes anxiety. In addition, personality traits related to being more pessimistic and anxious is enhanced in ASD [17]. Thus, there are reasons to believe that dental anxiety could be common in adults with ASD but to our knowledge this has so far not been investigated.

The aims of the present study were twofold, first to investigate if people with ASD have more negative dental experiences compared to a matched control group composed of general dental patients. The second aim was to compare the level of dental anxiety between the two groups.

## 2. Material and Methods

### 2.1. Study Setting

The study is a double-cohort study, where a group of adults diagnosed with ASD is compared to a group of typically developing adults. It comprises part of a study on oral health and dental experience in adults with ASD living in the northern Stockholm area in 2008. All participants attended a dental examination appointment and completed questionnaires. Then, they were provided a written and verbal report on their dental status and necessary dental treatment. Written consent was obtained from the participants, and ethical approval was obtained from the regional ethical board in Stockholm, Sweden (2008/874-31/4).

### 2.2. Participants

Forty-seven adults with ASD and of normal intellectual performance, and 69 age and sex-matched typically developing controls were included in the study. Exclusion criteria for all participants were diagnosis of intellectual disability, a history of brain damage, current or past neurological disorder, epilepsy, alcohol abuse or dependence, past or present substance abuse, and psychosis. In addition, scores above cutoff for a probable ASD, according to a rating scale for assessing autistic traits (described below), were an exclusion criterion for the controls. Demographic characteristics and descriptive statistics are presented in Table 1.

### 2.3. Participants with ASD

The participants with ASD were recruited from tertiary units for diagnosing ASD in adulthood, the Neuropsychiatric unit, Northern Stockholm psychiatric clinic, and from a community-based unit for adults with ASD located in Stockholm. The diagnosis of ASD was based on a thorough examination, which took 12–18 hours to complete, during a time period of two weeks to three months. The diagnostic assessment included structured and semistructured clinical interviews following the DSM-IV-TR [13] criteria for autistic disorder, Asperger disorder, or PDD-NOS, rating scales and an interview with a parent. In addition, a licensed psychologist performed neuropsychological tests including the Wechsler Adult Intelligence Scale-Revised (WAIS-III-R). A senior psychiatrist and the psychologist, both trained in diagnosing ASD, performed all assessments and the diagnosis was established in consensus between the two, but the psychiatrist was ultimately responsible for setting the diagnosis.

An invitation letter was sent to patients diagnosed with ASD at the Northern Stockholm psychiatric clinic and to subjects with ASD who were registered at the community-based unit. Sixty-nine subjects with ASD agreed to participate by a letter of reply; fifty-nine of these completed a telephone interview. Seven patients were unavailable for the dental appointment and another five failed to attend, resulting in a final study group comprising 47 adults with ASD (25 males, 22 females).

### 2.4. Controls

Sixty-nine (34 males, 35 females) typically developing adults were recruited from six dental clinics in the Stockholm area, after being asked during their dental examinations. They were given the same invitation letter as the ASD group. The control group was matched to the clinical group with regard to age, gender, and area of residence.

### 2.5. Demographic and Background Information

Demographic and background information was collected from the participants in conjunction with the dental appointment, using a self-administered questionnaire comprising questions on medical diagnosis, medication, country of birth, education of parents, educational level, occupational status, and tobacco use.

### 2.6. Screening for ASD

In order to screen for behaviours related to ASD and thereby disclosing any subjects with symptoms of ASD from the control group, all participants completed the autism spectrum quotient, AQ [18]. The AQ is a self-rating scale that describes a variety of traits typically observed in individuals with ASD. It consists of 50 items, assessing personal preferences and habits. Subjects rate to what extent they agree or disagree with the statements on a 4-point Likert scale, ranging from definitely agree (0) to definitely disagree (3). Items are subsequently coded dichotomously into 0 and 1 to reflect the absence or presence of each symptom. Total AQ scores reflect the sum of all items; the lowest possible score (i.e., 0) indicates no autistic traits and the highest possible score (50) indicates severe autistic traits. A score above 32 has been proposed as a cutoff score for probable ASD [18], but people with a confirmed ASD diagnosis sometimes have lower scores [19].

### 2.7. Dental Experience

All participants completed a questionnaire on previous dental experiences included for the purpose of this study. If they had visited the dentist after 20 years of age (dental appointments are free of charge in Sweden until 20 years of age), the reasons for the appointment were requested (dental pain or for other reasons unrelated to pain). The four following items were (1) pain experience during dental treatment, (2) insufficient dental anaesthesia, (3) pain during other medical treatments, and (4) feelings of being forced into dental treatment that they were not prepared for. Each item could be answered by the alternatives “never,” “once,” or “more than once”; the items are shown in Table 2. The first three of these questions have previously been used in a Norwegian study on dental anxiety [4]. Responses reflecting “The answers never” and “once” were combined into one group versus “more than once” in the statistical analysis.

### 2.8. Dental Anxiety

The Dental Anxiety Scale (DAS) [20] is the most widely used test for measuring dental anxiety and is filled out by the patient. The primary focus of the scale is on the anticipation of dental treatment. The DAS comprises four multiple-choice questions addressing the individual's reactions and expectations of going to and being treated by a dentist (anticipation; waiting in a waiting room; waiting for the drill; waiting for the scraping; the items are shown in Table 2). Each question consists of five response alternatives ranging from 1 (no anxiety) to 5 (extreme anxiety) with a total score varying between 4 and 20. Average DAS scores of 8-9 for ordinary (nonfearful) patients and ≥13 among fearful dental patients have been reported [20–22]. The CDAS has shown good validity in Swedish studies on patients with dental fear [22, 23].

The Revised Dental Beliefs Survey, DBS-R [2] assesses the patient's subjective perceptions regarding the dentist's behaviour and the process of how the care is delivered and issues that are of primary concern to patients with general anxiety or who are distrustful [2]. The instrument consists of 28 items with a 5-point Likert scale and a sum of score ranging from 28 to 140. The DBS-R has been found to be a reliable and valid instrument for use in various Swedish patient and nonclinical population groups to assess attitudes to dentists and dental care [24]. DBS-R scores with a mean of 42 in ordinary patients and 88 among fearful dental patients have been reported [24].

### 2.9. Statistical Analysis

Mean scores were calculated for each rating scale. The scores for each rating scale were first summed to a total score and then divided by the number of items to which the patient had responded. Missing data were analyzed by multiplying the mean item score for each response by the number of items in each scale, thus obtaining comparable scores. This method was chosen after it had been ascertained that no statistically significant differences occurred between the overall mean scores calculated in this way and the overall mean scores obtained using only complete questionnaires. A response rate above 80% was required in each questionnaire to be included.

Mann-Whitney *U* test was used to determine the significance of differences between continuous variables for the two groups, except for age and AQ, where Student's *t*-test was used. Categorical data were compared using the chi-square test. If the observed frequency was below five in one cell Fisher's exact test was used. The statistical software SPSS version 21.0 was used for statistical analyses.

## 3. Results

Results on questions regarding previous dental experiences are presented in Table 3. Significantly more patients with ASD had experienced pain during dental as well as other medical treatments compared to the typically developing adults (Table 3). Twenty-two percent (10/46) of the patients with ASD felt they had been forced into dental treatment they were not prepared for several times compared to only 3% (2/67) in the control group (*P* = 0.003).

All patients filled out the DAS and DBS-R. The ASD group reported more dental anxiety than the control group, according to the DAS and DBS-R (Table 4). DAS ≥ 13 was reported for 7/47 (15%) in the ASD group compared to 4/69 (6%) in the control group.

Participants with ASD who reported recurrent pain during dental treatment, insufficient local anesthesia or a feeling of being forced to dental treatment one was not prepared for, scored higher on DAS than those with ASD with one or no such experience (DAS pain dental treatment 9.8 ± 3.9 versus 7.0 ± 2.7 (*P* = 0.022); DAS insufficient anesthesia 11.1 ± 3.8 versus 7.2 ± 3.1 (*P* = 0.045); DAS being forced 11.6 ± 4.8 versus 8.2 ± 3.2 (*P* = 0.030)). However no association between DAS and pain during other medical treatments was found. In the controls no association between recurring negative dental or medical experience and higher DAS scores was found.

Table 5 shows separate item mean scores and ranking order of the DBS-R in the ASD and control groups. In both groups, the three highest ranked items were item number 8 “When a dentist seems in a hurry I worry that I am not getting good care,” item number 23 “Once I am in the chair I feel helpless (things are out of my control),” and item number 21 “When I am in the chair I do not feel like I can stop the appointment for a rest if I feel in the need.” In the ASD group, this was followed by item number 26 “Dentists often seem in a hurry, so I feel rushed,” item number 3 “I worry if the dentist is technically competent and is doing quality work,” and item number 11 “I am concerned that dentists might not be skilled enough to deal with my fear or my dental problems,” whereas the same items were ranked as numbers 16, 23, and 16 in the control group.

Only one subject in the ASD group had no dental contact, in addition another 7 had only booked an appointment when in dental pain, all others reported having a regular dental contact. When comparing the 7 patients with ASD that had only booked a dental appointment due to pain to the 39 with ASD that had regular dental contacts, there were no differences in mean values of DAS or DBS-R.

## 4. Discussion

This study shows that adults with ASD report more painful dental experiences and more often feel they have been forced to dental treatment that they were not prepared for, compared to typically developing people. The results also show that adults with ASD experience greater dental anxiety and emphasize different aspects of dental anxiety.

The great majority of the participants in the ASD group felt pain several times during dental treatment compared to less than half of the control group. Pain due to insufficient local anaesthesia was experienced several times by twice as many in the ASD group compared to the control group. The patients in the ASD group may have experienced more pain because of abnormal perception or other sensory sensitivities as both hyper- and hyposensitivity are frequently reported in ASD [16]. The patients in the ASD group might also have a lowered pain threshold for some qualities of pain. During dental treatment under general anaesthesia the need of anaesthetic medication (the propofol requirement) was found to be greater amongst patients with autism than intellectually impaired controls [25].

The presumably accompanied heightened stress level may have enhanced the experience of pain [26]. The sense of pain is also influenced by dental anxiety, as dental anxiety is a reinforcer of pain [8]. Participants with high dental anxiety and high pain sensitivity expect and experience more pain during dental treatment [27]. On the other hand, painful experiences during dental treatment increase the risk for future dental anxiety. A Norwegian study showed that 18-year-old participants who reported more than one previous experience of pain during dental treatment were ten times more likely to report high dental anxiety compared to those without this experience [6]. In the present study, those who had experienced recurrent pain or recurrent insufficient anaesthesia during dental treatment reported more dental anxiety in the ASD group, but not in the control group. This finding could be explained by sensory overresponsivity in the ASD group, which presumably can cause anxiety by mechanism of conditioning [28]. The characteristic smell of a dental office or the sound of the drill could become conditioned stimuli resulting in anxiety. However, in the present study there was no association between recurrent painful medical treatments and dental anxiety in either of the groups, suggesting that the association with dental anxiety does not extend to settings outside the dental office.

The ASD group reported that they more often felt they had been forced to dental treatment they were not prepared for than the control group. In the ASD group, but not among the controls, recurrent experiences of “being forced to dental treatment one was not prepared for,” were associated with higher levels of dental anxiety. Across different cultures fearful patients put a strong emphasis on dentists' behaviour and performance of the dental treatment: emphatic behaviour and pain-free treatment are most important, while giving information and control are rated second [29]. Patients with ASD are impaired in predicting future events and have a low ability to build flexible predictions [16]. Thus the patient with ASD may feel completely un-prepared although others would think they were just as well prepared as any dental patient in a similar situation. Children with Asperger syndrome scored significantly lower on a language subtest on comprehension of instructions, while other subtests in the language tests showed no differences compared to typically developed controls [30] illustrating the risk of overestimating comprehension abilities in individuals with ASD.

The ASD group reported more dental anxiety compared to the control group. The control group scored lower than a comparative group of non-ASD Swedish patients, (mean DAS scores 8.0 and DBS-R scores 42.0) [24]. However, our ASD group reported higher scores, and the differences between the groups were marked when mean item scores were calculated: in the ASD group the mean DAS score was 1.8, in our control group 1.2 and in the Abrahamsson sample 1.5 [24]. The relatively low DAS scores in the control group could partly be due to the fact that they were familiar with their dentists, which may have reduced the anxiety level. On the other hand, high scores, reflecting dental anxiety, were also reported by some of the controls in the present study.

An intriguing finding was the different orders in ranking of items in the DBS-R between the groups. The group with ASD emphasized different aspects of dental anxiety than the control group. Although the three highest ranked items “When a dentist seems in a hurry I worry that I am not getting good care,” “Once I am in the chair I feel helpless,” and “When I am in the chair I do not feel like I can stop the appointment for a rest if I feel in the need” were identically ranked in the ASD and control groups, consistent with rankings in an earlier report [24], the items ranked as fourth, fifth, and sixth differed between the groups. These next three ranked items “Dentists often seem in a hurry, so I feel rushed,” “I worry if the dentist is technically competent and is doing quality work,” and “I am concerned that dentists might not be skilled enough to deal with my fear or my dental problems” were only ranked as numbers 16, 23, and 16 in the controls. Hence, the patients with ASD emphasized different aspects of dental fear compared to healthy controls (i.e., “dentist not technically competent, not skilled enough, I feel rushed”). Our findings suggest that people with ASD have a hard time trusting the professionals' ability to perform high-quality work, possibly related to deficits in mentalizing, a core symptom in ASD.

A sense of coherence (SOC) is defined by Antonovsky [31] as a pervasive feeling that (1) the stimuli deriving from one's internal and external environments are structured, predictable, and explicable; (2) resources are available to meet the demands posed by these stimuli; and (3) these demands are challenges, worthy of investment and engagement. According to a study on adolescents, those with higher dental fear have a weaker SOC than those with less dental fear [32]. In adult ASD extremely low SOC scores have been reported [33], but unfortunately SOC was not measured in our participants.

The DBS-R was constructed to assess to what degree patients perceive the interpersonal relationship with the dental professional as being the problem or possibly contributing to dental anxiety [2]. A change towards a more positive attitude in dentists' communication style early in the treatment has shown to predict a successful outcome in treatment of dental fear [34]. The communication impairment, critical for the diagnosis of ASD, suggests that specific communication skills are required from dental personnel, in order to meet the needs of the ASD population.

Dentists use a variety of techniques to meet the needs of fearful patients [35]. One of the most effective approaches to reducing dental anxiety is optimising controllability and predictability for the patient, by providing the patient with accurate explanations and information about what will happen and what type of experiences she/he might expect [36]. As ASD involves impairment in communication and sensory oversensitivity, this is particularly important. The latter is explained by the fact that people with ASD process visual and other stimuli differently presumably due to poor connections between parts of the brain that process these stimuli [37]. Thus the dentist should carefully inform the patient with ASD what she/he is about to do and what it may feel like, in addition to slowing down the pace of speech which facilitates comprehension. Another option is the use of visual pedagogy. Visual pedagogy is a way of introducing dentistry to children with ASD. When visual pedagogy was used to introduce dentistry to children with ASD, the capacity of children to cooperate during dental treatment one and a half year later was superior to that of children with ASD who had not been offered visual support [38]. In a recent study on adults with ASD and mild intellectual disability, the cooperation during a dental examination was significantly increased after a five-session training programme using a TEACCH-based approach [39]. Also, there is a growing interest in cognitive behavioural therapy for treating anxiety in adult ASD [33, 40]. The method is known to be helpful for treating dental anxiety in typically developing adults [1] but has not been studied for treating dental anxiety in people with ASD. Therefore, interventional studies on dental anxiety in ASD are warranted.

A limitation of the current study was the dropout in the ASD group due to patients not showing up at the dental appointment. The results regarding the ASD group might also not be representative of all persons with ASD, as the patients chose themselves to participate in the study. The present study assesses dental experiences and dental anxiety, known to be associated with dental avoidance [2]. Individuals who chose not to participate in the study or did not show up at the appointment probably included persons with high levels of dental anxiety. Dentally anxious persons, who believe they have bad oral health, tend to avoid going to the dentist [41]. Therefore, if persons with ASD and high dental anxiety chose not to participate in our study, this would have attenuated the differences in dental anxiety from the controls, which would further strengthen our findings. Another limitation was that as the control group consisted of regular dental patients recruited from dental practices, in this group we did not capture irregular attendees or the avoiders. Irregular attendees are more likely to be very afraid of visiting the dentist than regular attendees [42]. Levels of dental anxiety, general anxiety, and depression are shown to be higher among irregular attendees compared with regular attendees [43]. The vast majority (83%) amongst our ASD group had a regular dental contact and, notably, there were no differences regarding the anxiety between those with ASD who had a regular contact and those who did not.

## 5. Conclusion and Clinical Implication

In conclusion, people with ASD reported more painful dental experiences and more often felt they were forced to dental treatment they were not prepared for, they have more dental anxiety and emphasize different aspects of dental anxiety compared to typically developing people. Dental treatment and methods for supporting the communication with patients with ASD need to be developed, in order to reduce the negative dental experiences and dental anxiety in people with ASD.

## Figures and Tables

**Table 1:** Sociodemographic characteristics of adults with autism spectrum disorder (ASD) and controls.

	ASD group	Control group	*P*-value
	*n* = 47	*n* = 69	
Mean age (years)	33 ± 8	34 ± 7	0.466^*^
Male sex, *n* (%)	25 (53)	34 (49)	0.679^#^
Mother's country of birth *n* (%)			
Sweden	39 (83)	57 (83)	
Other Nordic countries	4 (9)	5 (7)	
Other European countries	1 (2)	2 (3)	
Rest of the world	3 (6)	5 (7)	0.985^#^
Father's country of birth, *n* (%)			
Sweden	36 (77)	59 (86)	
Other Nordic countries	5 (11)	3 (4)	
Other European countries	3 (6)	2 (3)	
Rest of the world	3 (6)	5 (7)	0.441^#^
Highest educational level, *n* (%)			
Elementary school	3 (6)	2 (3)	
High school	28 (60)	25 (36)	
University/college	16 (34)	41 (59)	0.021^#^
Working or studying, *n* (%)			
Full time	11 (23)	63 (91)	
Part time	7 (15)	5 (7)	
Unemployed/not studying	29 (62)	1 (1)	<0.001^#^
Smoking, *n* (%)	2/47 (4)	9/69 (13)	0.195^a^
Snuff, *n* (%)	4/47 (9)	8/69 (12)	0.759^a^
Medication, *n* (%)	31/47 (66)	7/69 (10)	<0.001^#^
Medication with reported side-effect xerostomia, *n* (%)	23/47 (47)	1/69 (1)	<0.001^#^
AQ (range)	133.8 ± 25.9 (66.0–182.8)	95.1 ± 11.1 (75.0–128.0)	*P* < 0.001^*^
AQ single count (range)	28.7 ± 10.4 (5.0–46.0)	11.4 ± 4.5 (4.0–28.0)	*P* < 0.001^*^

^*^ *t*-test.

^#^ *χ* ^2^ test.

^a^Fisher's exact test.

**Table 2:** Corah Dental Anxiety Scale.

Estimate the level of fear you would perceive in the situations described below.	
(1) If you had to go to the dentist tomorrow, how would you feel about it?	
(a) I would look forward to it as a reasonably enjoyable experience.	
(b) I would not care one way or the other	
(c) I would be a little uneasy about it.	
(d) I would be afraid that it would be unpleasant and painful.	
(e) I would be very frightened of what the dentist might do.	
(2) When you are waiting in the dentist's office for your turn in the chair, how would you feel?	
(a) Relaxed.	
(b) A little uneasy.	
(c) Tense.	
(d) Anxious.	
(e) So anxious that I sometimes break out in seat or almost feel physically sick.	
(3) When you are in the dentist's chair waiting while he gets his drill ready to begin working on your teeth, how do you feel?	
(a) Relaxed.	
(b) A little uneasy.	
(c) Tense.	
(d) Anxious.	
(e) So anxious that I sometimes break out in seat or almost feel physically sick.	
(4) You are in the dentist's chair to have your teeth cleaned. While you are waiting and the dentist is getting out the instruments, which he will use to scrape your teeth around the gums, how do you feel?	
(a) Relaxed.	
(b) A little uneasy.	
(c) Tense.	
(d) Anxious.	
(e) So anxious that I sometimes break out in seat or almost feel physically sick.	

**Table 3:** Dental experiences in autism spectrum disorder (ASD) and controls.

More than one experience of	ASD	Control	*χ* ^2^	*P* value
	*n* (%)	*n* (%)		
Pain during dental treatment^a^	33/46 (70)	30/67 (45)	6.8	0.009^#^
Insufficient local anaesthesia	20/47 (43)	14/67 (21)	14.7	0.001^#^
Pain during other medical treatment	27/47 (57)	11/67 (16)	20.9	<0.001^#^
A feeling of being forced to dental treatment one was not prepared for	10/46 (22)	2/67 (3)	10.1	0.003^a^

^#^ *χ* ^2^ test and ^a^Fischer's exact test.

**Table 4:** Dental anxiety and beliefs in autism spectrum disorder (ASD) and controls.

	ASD	Control	*P*-value
	*n* = 47	*n* = 69	
DAS, mean score (SD)	8.9 (3.8)	6.9 (2.8)	0.003
DAS, median score (range)	8 (4–19)	6 (4–18)	
DBS-R, mean score (SD)	50.1 (20.5)	34.3 (10.7)	<0.001
DBS-R, median score (range)	46 (28–108)	31 (28–89)	
DBS-R, mean item score (SD)	1.8 (0.7)	1.2 (0.4)	<0.001
DBS-R, median item score (range)	1.6 (1.0–3.9)	1.1 (1.0–3.2)	

DAS: Corah Dental Anxiety Scale.

DBS-R: Revised Dental Beliefs Survey.

**Table 5:** Item mean scores and ranking of Dental Beliefs Survey (DBS-R) in autism spectrum disorder (ASD) and controls.

Items	ASD	Rank	Control	Rank	*P* value^#^
	*n* = 47 Mean (SD)		*n* = 69 mean (SD)		
(1) I am concerned that dentists recommend work that is not really needed	1.7 (1.0)	19	1.3 (0.7)	5	0.029

(2) I believe dentists say/do things to withhold information from me	1.3 (0.8)	28	1.1 (0.3)	27	0.022

(3) I worry if the dentist is technically competent and is doing quality work	2.0 (1.2)	5	1.1 (0.5)	23	<0.001

(4) I have had dentists say one thing and do another	1.5 (0.9)	24	1.3 (0.7)	10	n.s.

(5) I am concerned that dentists provide all the information I need to make good decisions	1.8 (1.3)	14	1.1 (0.3)	26	<0.001

(6) Dentists do not seem to care that patients sometime need a rest	1.9 (1.2)	11	1.2 (0.6)	10	0.001

(7) I have had dentists seem reluctant to correct work unsatisfactory to me	1.7 (1.3)	19	1.3 (0.6)	8	n.s.

(8) When a dentist seems in a hurry I worry that I am not getting good care	2.5 (1.4)	1	1.6 (1.0.)	1	<0.001

(9) I am concerned that the dentist is not really looking out for my best interests	1.9 (1.3)	9	1.1 (0.5)	16	<0.001

(10) Dentists focus too much on getting the job done and not enough on the patients comfort	1.9 (1.2)	8	1.3 (0.6)	9	0.001

(11) I am concerned that dentists might not be skilled enough to deal with my fear or my dental problems	2.0 (1.3)	6	1.1 (0.6)	16	<0.001

(12) I feel dentists do not provide clear explanations	1.8 (1.1)	14	1.2 (0.5)	13	0.001

(13) I am concerned that the dentists do not like to take the time to really talk to the patients	1.8 (1.1)	14	1.2 (0.5)	14	<0.001

(14) I feel uncomfortable asking questions	1.8 (1.2)	13	1.1 (0.5)	16	<0.001

(15) Dental professionals say things to make me feel guilty about the way I care for my teeth	1.7 (1.0)	19	1.4 (0.7)	4	n.s.

(16) I am concerned that the dentists will not take my worries (fears) about dentistry seriously	1.7 (1.2)	17	1.1 (0.5)	23	<0.001

(17) I am concerned that the dentists will put me down (make light of my fears)	1.3 (0.6)	27	1.1 (0.3)	27	0.003

(18) I am concerned that the dentists do not like it when a patient makes requests	1.9 (1.2)	9	1.2 (0.6)	14	<0.001

(19) I am concerned that the dental personnel will embarrass me over the condition of my teeth	1.4 (1.0)	26	1.1 (0.4)	22	n.s.

(20) I believe that dentists don't have enough empathy for what it is really like to be a patient	1.5 (0.8)	23	1.1 (0.3)	25	<0.001

(21) When I am in the chair I don't feel like I can stop the appointment for a rest if I feel in the need	2.3 (1.4)	3	1.4 (0.9)	3	<0.001

(22) Dentists don't seem to notice that patients sometime need a rest	1.9 (1.1)	7	1.3 (0.7)	6	<0.001

(23) Once I am in the chair I feel helpless (that things are out of my control)	2.4 (1.4)	2	1.6 (0.8)	2	0.004

(24) If I were to indicate that it hurts, I think that the dentist would be reluctant to stop and try to correct the problem	1.8 (1.2)	12	1.1 (0.5)	16	<0.001

(25) I have had dentists not believe me when I said I felt pain	1.6 (1.0)	22	1.2 (0.6)	12	0.021

(26) Dentists often seem in a hurry, so I feel rushed	2.1 (1.3)	4	1.1 (0.4)	16	<0.001

(27) I am concerned that the dentist will do what he wants and not really listen to me while I am in the chair	1.7 (1.1)	18	1.1 (0.4)	16	0.002

(28) Being overwhelmed by the amount of work needed (all the bad news) could be enough to keep me from beginning or completing treatment	1.5 (1.1)	25	1.3 (0.8)	7	n.s.

^#^Mann-Whitney *U* test, comparing the mean values.
